# Comparing the role of neuronal versus non-neuronal cells in the pathophysiology of delirium: a systematic review

**DOI:** 10.1192/j.eurpsy.2022.1817

**Published:** 2022-09-01

**Authors:** H. Mcadam

**Affiliations:** University of Edinburgh, Edinburgh Medical School, Edinburgh, United Kingdom

**Keywords:** pathophysiology, systematic review, neuronal, delirium

## Abstract

**Introduction:**

Delirium is a condition which impacts nearly half of older adults during hospital admission. It presents with a wide range of neuropsychiatric symptoms leading to increased morbidity and mortality. Despite this, specialised knowledge and ownership of the condition remain unclear.

**Objectives:**

To compare evidence surrounding the roles of neuronal and non-neuronal cells in the overall pathophysiology of delirium and consider the impact this could have in practice.

**Methods:**

Using PRISMA systematic review guidelines, five medical research databases were screened for papers discussing the role of neuronal and/or non-neuronal cells in the pathophysiology of delirium between 2011 and 2021.

**Results:**

Fifteen papers which met the inclusion criteria were then categorised into discussing neuronal (n=2), non-neuronal (n=4) or both (n=9) types of cells’ roles in the pathophysiology of delirium. Delirium was often caused by a homeostatic imbalance secondary to acute illness leading to deterioration of neural synapses and therefore signal transmission. However, it was also argued that activated non-neuronal cells, particularly microglia and astrocytes, played a significant role through disruption of the blood brain barrier. This was likely to play a role in the more severe clinical presentations of delirium.

**Conclusions:**

The pathophysiology of delirium is multifactorial with neuronal and non-neuronal cells implicated in neurological disruption. There is no clear agreement on how these mechanisms vary according to aetiology and, ultimately, the severity of delirium. Further research will help refine these theories, which will support the pharmacological and clinical management of the condition.

**Disclosure:**

No significant relationships.

